# Interaction between Nursing Staff and Residents with Aphasia in Long-Term Care: A Mixed Method Case Study

**DOI:** 10.1155/2018/9418692

**Published:** 2018-12-02

**Authors:** Charlotta Saldert, Hannah Bartonek-Åhman, Steven Bloch

**Affiliations:** ^1^Institute of Neuroscience and Physiology, Division of Speech-Language Pathology, Sahlgrenska Academy, University of Gothenburg, Gothenburg, Sweden; ^2^University of Gothenburg Centre for Person-Centred Care (GPCC), Sahlgrenska Academy, University of Gothenburg, Gothenburg, Sweden; ^3^Language and Cognition, University College London, UK

## Abstract

**Introduction:**

Thousands of individuals with communication disorders live in long-term residential care. Nursing staff are often their primary communication partners. The positive effects of social interaction and person-centred care have been recognised but there remains a paucity of research on the content and quality of communicative interaction between long-term care staff and residents with aphasia. This mixed method study investigates the discourse in interaction between nursing staff and residents with aphasia.

**Methods:**

A routine care activity was explored in 26 video-recordings featuring four enrolled nurses and four elderly persons with severe aphasia. Factors such as goals and roles in the activity were mapped out and a qualitative discourse analysis was performed. Based on the findings a coding scheme was constructed and the amount of time spent in different interactional foci of discourse was explored.

**Results:**

From the qualitative findings three broad, but distinct, foci in the nurse-initiated interaction could be distinguished: (1) a focus on getting the task done with minimum interaction; (2) topics related to the task, but not necessary to get the task done; and (3) personal topics related to themes beyond the caring task. The analysis of distribution of time revealed that although most of the interaction was focused on the main care activity, between 3 and 17% of the time was spent in either task-related or non task-related interaction. The distribution varied between dyads and could not be related to the residents' severity of aphasia nor the activity as such.

**Conclusions:**

An endeavour to interact socially with the residents with aphasia influences the nurses' foci of interaction. Contextual and personal factors of the residents and nurses need to be considered in clinical work as well as research on how communication may be supported to facilitate social interaction and person-centredness in long-term care of people with aphasia.

## 1. Introduction

Due to demographic changes and progress in medical care, an increasing number of elderly people are living with physical disabilities and communication disorders due to neurological disease or injury. Communication disorders, such as aphasia following a stroke, may severely affect a person's ability to understand and convey information in speech and writing, as well as the ability to interact socially. Numerous individuals, who are in need of more assistance than can be delivered by home care services, are living in long-term care facilities [1]. In this context communication difficulties pose an additional challenge [2].

An endeavour to humanise medicine and care has been described, with both similarities and differences under different labels such as client-centred care [3], patient-centred care [4, 5], person-centred nursing [6], person-centred care [7, 8], or person-centredness [9]. In person-centred care the individual person beyond the role of being a patient or a resident with needs is acknowledged. Each individual's personal experiences and traits are recognised and considered in the planning and achievement of care. Functional communication in the encounters between nursing staff and residents in long-term care facilities is clearly a prerequisite to accomplish this. In long-term care, person-centred care has been described in the care of people with communication disorders due to dementia and it has been proved to reduce both disruptive behaviour and need for medication [10–13].

The importance of communication is often emphasised when staff in long-term residential care describe what they consider to be good caring encounters [14, 15]. Being able to have conversations with residents makes it possible to get to know each person's unique life history, to regard them as individuals and to establish and develop personal relationships. It is also known that meaningful social interaction is essential for the wellbeing of residents in long-term care facilities [16]. Staff attitudes are signalled in the interaction and affect the residents' perception of quality of life. Furthermore, many elders living in long-term care facilities feel their needs are often ignored by the staff and they also stress the lack of communication beyond instrumental interactions. The fact is that although nursing staff acknowledge the importance of social interaction with the residents, long-term care facilities have been described as providing limited possibilities for communication outside of care routines [14, 15, 17].

Some attempts to describe the different foci of interaction in different care contexts have been made quantitatively. Roter and Hall [18] described two purposes of communication between doctors and patients during medical visits: (1) instrumental or task-focused or (2) affective or rapport-developing. Although they are not necessarily mutually exclusive, a distinction between task-focused interaction and more affective interpersonal interaction focusing on more general personal issues is often reflected in research on interaction between nursing staff and patients or residents. One example is Bottorf and Morse's [19] qualitative observational video-recording analysis of registered nurses attending care of cancer patients. Besides exploring different types of touch, four types of attending were identified and described: (1)* Doing more*: making contact or the nurse “did something” beyond what is required to complete the care; (2)* Doing for*: primarily responding to patient requests and needs that were not treatment related; (3)* Doing with*: focus equally on the task and patient, for example, the nurse may have actively engaged a patient by seeking or attending to his or her opinions, thoughts, and perceptions; (4)* Doing tasks*: focus on equipment, treatment, and getting the job done. The authors conclude that the dynamic quality of nurse-patient interaction is not only an internal experience, but one that can be observed in verbal and nonverbal behaviours of nurses and patients.

Previous research in the context of long-term care has reported an overemphasis on task-focused talk and lack of opportunities for more personal psychosocial or affective interaction [20–22]. Williams and colleagues [17] examined topics of staff-resident interaction. A scheme based on previous research was developed to categorize topics of staff talk with residents. About thirty-nine percent of the utterances focused on activities of daily living (ADLs), 14% focused on assessment of nursing, 16% focused on technical aspects of care, and 29% were more personal, psychosocial in nature.

Based on Roter and Hall's [18] conceptualising, four characteristics of affective interaction have been described in verbal communication between nurse aides and residents in a long-term care facility [23]: (1) personal conversation (including pleasantries, laughter, and conversation about the resident's personal life); (2) addressing the resident by name or use of terms of endearment (like “honey” and “sweetie”); (3) checking in (asking residents whether they were all right or about their comfort level in the task); and (4) emotional support/praise. Still, the authors do say that this affective communication was sometimes also used by the nurse aides in instrumental communication to improve the effectiveness of their care delivery. The purpose of nurse aides' communication varied depending on the cognitive functioning of the residents. With residents diagnosed with more severe dementia, the nurse aides asked fewer questions and instead made more imperative statements characterized as being more instrumental than affective by referring to the current care activities.

Topics in first morning encounters between nurses and nursing assistants and elderly residents in long-term care have been investigated [23]. It was concluded that it was the staff who initiated conversation and they also chose the topic of conversation, which was usually regarding residents' health and sickness. The fact that it is usually staff who initiate and control the topic of conversation has also been described elsewhere [14, 22].

Attempts have been made to develop instruments for assessing degree of person-centredness in communication [24–26]. Savundranayagam and colleagues have developed a scheme for coding frequency of use of person-centred communicative strategies with people with communication difficulties due to dementia [27–29]. In this instrument a nursing staff's use of communicative strategies is quantified without considering the context of the communicative interaction. This neglect of the influence of context on communicative interaction is actually the case in most of the existing research on communication between nursing staff and the elderly in long-term care and affects the validity of the results [30, 31]. For example, in the first morning encounters, as described in Wadensten [23] the staff member is entering the resident's room to help them get out of bed, getting dressed, etc. The external context of this activity affects the roles, the behaviours, and the discourse of the participants in the interaction. However, there is also an internal context within a communicative interaction on a sequential level where each contribution or behaviour is affected by previous contributions and also influences the contributions that will follow [32].

Marsden and Holmes [31] have questioned the inferences from earlier research on health care providers' communication with elderly people as this usually has not considered aspects of coconstruction of the interaction and, for example, the face-saving actions in everyday interactions.

Conversation analysis (CA) is a qualitative, data driven method used to study naturally occurring interaction and the collaborative accomplishment and organization of social action [33]. The method has been used in numerous studies of conversational interaction in different institutions including interaction between staff and residents in long-term care [34–36]. CA has also been used to explore interactions between nursing staff and people with dementia in long-term care [22, 37, 38]. However, although CA focuses on the internal sequential context of talk, its ethnomethodological theoretical base stipulates that external factors which are not oriented to by the participants themselves should not be considered [39].

By contrast, the method* Activity-based Communication Analysis* (ACA) emphasises the influence from external context on the interaction [40, 41]. ACA is a multidisciplinary framework based on philosophical and linguistic as well as psychological and sociological theories in the view of language as action in context [42–45]. In ACA the outset for the analysis is the type of activity where the interaction occurs. ACA treats aspects such as participants' roles and goals in the social activity as relevant factors, influencing the interaction. The communication difficulties seen in communication disorders have been important in the development of the model [46, 47]. According to ACA the collective influencing factors, for example, the main goal of the activity, the integral roles of the participants, and physical circumstances, are common to all participants in the activity. These collective factors interact with the participant's individual background factors, including, for example, their physical and cognitive competencies, and ability to use different means to produce and comprehend utterances. Participants in communication may also have individual goals and take on different roles beyond those inherent in the specific activity. The influencing factors of the activity and participating individuals interact in determining the communicative interaction that actually takes place.

To summarize, many individuals with severe aphasia are living in long-term residential care and the importance of social interaction and person-centred care for residents' experience of quality of life has been acknowledged. Research has shown that routine care activities may be more or less task-focused and include different degrees of social interaction. However, there is still a lack of evidence regarding interaction between nursing staff and long-term care residents with aphasia based on analysis that considers both external and internal sequential contextual factors.

The purpose of this study was to explore the discourse and interactive patterns of nursing staff working with people with severe aphasia in a routine care task in long-term care facilities. The aims were to (1) explore contextual factors affecting the communicative interaction between enrolled nurses and residents with severe aphasia; (2) describe the nurses' interactional foci in their work with the persons with aphasia during a routine care activity.

## 2. Materials and Methods

This sequential exploratory mixed method study has been approved by a regional ethical review board and all participants provided informed written consent.

### 2.1. Participants

The participants in this study were recruited through the managers and staff of different long-term residential care facilities in western Sweden and are a convenience sample. Four dyads comprising a person with stroke-induced aphasia and an enrolled nurse participated in the present study; see Table 1. An enrolled nurse in Sweden has completed a formal 65-week-long training program in nursing. They provide the routine care work under the supervision of a registered nurse.

Inclusion criterion for the nurses was regular contact with the person with aphasia. Inclusion criterion for the persons with aphasia was a presence of communication difficulties caused by stroke-induced aphasia. Exclusion criteria for both nurses and persons with aphasia were vision or hearing impairment not compensated for by aids. All participants were native Swedish speakers.

The participating nurses were all female with an age range of 36 to 55 years (see Table 1). They had worked with the resident with aphasia for between nine months to three years, but all had long experience in working with people with communication disorders. The nurses had between 11-12 years of education.

The comprehension of the participating persons with aphasia was measured with the Token test [48] and semantic and phonological word fluency was measured using standardised test procedures and scoring standards [49].

The participants with aphasia comprised one man and three women, between ten months and eight years after onset of aphasia and with an age range of 72 to 93 years (see Table 1). All four residents with aphasia had severe aphasia with incomprehensive speech and impaired comprehension. They were all able to express acceptance or rejection vocally, but their speech output was otherwise reduced into syllable repetitions together with neologistic words and phrases. All four residents also expressed themselves with facial expressions and variations of prosody.

### 2.2. Material: Video-Recordings of Naturally Occurring Interaction

The participants selected a routine nursing activity. In three of the dyads (1, 3, and 4) the participants chose to do the video-recording during the morning nursing routine task. Dyad 2 chose the evening routine. A research assistant set up the video camera and then left the dyads alone, except for dyad 1 where the assistant operated the camera throughout the recording. The dyads were instructed to interact as they usually would in that situation.

Each dyad was video-recorded between five and nine times. The length of each recording varied between approximately 10 and 25 minutes. The middle 10 minutes of each recording were used in the analysis based on the hypothesis that individuals are less self-conscious after being recorded for a few minutes.

### 2.3. Procedures

An analysis of the influencing and influenced factors in the main activity, including the present goals and roles, was performed in accordance with ACA [46]. This was followed by a discourse analysis of the video-recorded interaction [50]. The analysis was influenced by CA [33] in the exploration of interactional patterns on a sequential level, but mainly performed with the purpose of revealing the content and foci of the interaction between the nurses and residents. Using standard CA conventions, the video-recorded interactions were transcribed to capture talk as well as nonvocal features such as gestures and other body movements, (see Table 2 for transcription symbols used). The transcriptions presented here comprise representative extracts from the material and have been translated into English.

From the qualitative analysis it was concluded that the nurses' communicative interaction with the residents could be described as belonging to one of three broad but still distinctive categories of interaction with different goals and content (see results). As different qualities of touch, facial expressions, and gaze may also communicate meaning, the categories may include sequences both with and without verbal interaction.

A quantitative coding scheme was also constructed to enable an exploration of the distribution in time of the different types of interaction. The qualitative analysis showed that it was the nurses who decided whether to elaborate on a topic or to change focus in the interaction. The persons with aphasia only rarely initiated interaction and their verbal contributions were difficult to interpret due to the severe aphasia. Thus, the coding of the interaction across time was based on the start and ending of the nurses' actions.

In the procedure for analysis of distribution of time the primary assessor coded the content in the video-recordings using the annotation program ELAN (Max Planck Institute for Psycholinguistics, The Language Archive, Nijmegen, Netherlands). Each second in the video-recordings was coded as belonging to one of the three categories based on an assessment of what was the nurse's main focus of the interaction. The number of seconds allocated to each category was then calculated and presented as the proportion of time used for the different foci in each dyad's video-recordings.

The reliability of the coding scheme was explored in a large material of video-recordings with the participants. The analysis of intrajudge reliability was performed with calculation of point-to-point agreement on coding performed by the primary assessor (a speech-language pathologist and researcher, the second author) on two different occasions with at least one week but no more than two weeks between them. Intrajudge reliability was calculated on 42% of the material (833 codings), including recordings from all dyads, and exceeded 98% agreement. Interjudge reliability was calculated on 38% of the material (689 codings) and exceeded 90% agreement between the primary assessor's coding and coding done separately by a second assessor (a speech-language pathologist and researcher, first author).

## 3. Results

### 3.1. Activity-Based Communication Analysis

Results from an analysis of influencing and influenced factors in the interaction, including collective and individual goals, are presented below.

#### 3.1.1. Factors Inherent in the Main Activity

The main overarching activity in all video-recordings was a routine care task with the shared goal to get the residents ready for the day or in one case (dyad 2) for bed at night. This was also considered a shared goal in all dyads.

In all four dyads this main overarching activity contained similar care related components or subactivities, including moving between bed and bathroom; getting medicine; washing upper body; getting (un)dressed; brushing hair and teeth; and receiving face cream or a shave. The order of the components varied between the dyads but was usually the same each time within each dyad. In this main activity the role of the nurse was to help the resident to go through the whole procedure. The residents' role was to accept the help and cooperate in the subactivities in the routine care task.

Another shared goal was to take the opportunity to interact socially as this was usually the only time during the day where the residents and the nurses spent a little longer time together alone [14–17]. Attainment of this goal assumes that the participants are able and willing to take on other roles than those assumed by the main overarching care activity. Both the nurses and the residents are expected to take on the role of being a conversation partner with a shared interest in, and responsibility for, doing the best they can to maintain the social interaction.

#### 3.1.2. Individual Background Factors and Their Influence on the Activities, Roles, and Goals

The participating nurses were all experienced in working in long-term residential care with people with communication disorders. They had also worked with the participating residents for some time and had had the opportunity to get to know the residents' personal traits and preferences.

All four residents had stroke related hemiplegia affecting their ability to move their limbs, making it impossible for them to attend to their own personal care. In this routine activity they were referred to the role as a receiver of care, being dependent of the nurse. This role may result in a certain degree of passivity and lack of initiative in the interaction.

The residents had severe global aphasia which was an individual background factor affecting their ability to take on the role as conversation partner in the interaction with the nurses both in the physical routine care and in the social interaction.

### 3.2. Results from the Qualitative Analysis of the Interaction

The collective and individual background factors influenced the communicative patterns in the dyads in several ways.

In dyad 1 (see Extract 5) the nurse would initiate interaction by asking questions about the resident's experiences and preferences in the routine care activity or, for example, about how he had been since last time they saw each other or about his family. When mutual understanding was compromised, the nurse would sometimes ask for clarification but then abandon repair without producing any candidate solutions or suggestions. In her role as communication partner, the nurse also frequently told the resident about what happened in her own life since they last met and the resident contributed with verbal and nonverbal responses.

In dyad 2 the resident was the only one among the participating residents who often initiated communication although her speech was neologistic and incomprehensible. Although she took on the role of conversation partner she was not able to self-repair trouble related to her verbal contributions. The nurse would sometimes provide a candidate solution, but she also often abandoned the repair and instead shifted the topic or simply left the resident's utterances unattended. Despite the resident's incomprehensible speech, the nurse would often comment on or ask questions about the resident's experiences and wishes in the current care activity (see Extract 2). In her role as conversation partner, she would also often preserve the conversational flow by talking about activities she had participated in herself rather than asking the resident about non task-related issues.

The resident in dyad 3 would sometimes initiate speech but would typically only produce single mispronounced syllables and then give up. The nurse often used yes and no questions when interacting with the resident. They seemed to have a shared interest in beauty care and the nurse often asked the resident about her beauty products (see Extracts 3 and 4). The nurse's questions were usually delivered at a fast pace and although she sometimes acknowledged the resident's contributions, she would often move on in the conversation. Although the resident in dyad 3 would produce brief responses to the nurse's questions, she seldom tried to elaborate on the topics initiated by the nurse.

In dyad 4 the resident rarely took the initiative to communicate. The nurse frequently used yes and no questions and also delivered instructions at a fast pace before proceeding with the routine care task (see Extract 2). Neither of the participants in this dyad seemed to put too much effort in assuming their roles as conversation partners. The nurse rarely initiated communication about matters beyond the task at hand. Nevertheless, there was a lot of humour (laughter and smiles), especially on behalf of the nurse, in the interaction during the routine care task.

### 3.3. Three Different Foci of Interaction

The discourse analysis showed that the shared goal of social interaction between the nurses and the residents could be attained by a continuum of different types of interaction more or less related to the physical nursing task and involving different topics. Within this continuum, three broad, but distinct, categories of interaction could be distinguished: (1)* task-central interaction* where the main goal of the nurses was to get the task done; (2)* task-related interaction *where the goal of social interaction was worked on by involving the resident in the task. For example, by providing a choice or inquiring about the how residents' experienced the current tasks or about issues related to, but not concerning, the specific task at hand; and (3)* non task-related interaction, *where the nurses would bring up topics not related to the task at hand. Subactivities motivated by the goal to get the resident ready for the day/night may have continued but were not in the focus of the interaction during the subactivities aimed at social exchange. These categories are presented in five extracts from the transcribed video-recorded interaction (see Extracts 1–5).

**Extract 1: Task-central interaction (dyad 2): commenting on the task procedure.**
01N-2 * ((N gives R a spoonful of yoghurt))*
02⌈then that was finished
⌊((*scrapes up the remains in the cup with the spoon))*
03((*continues scraping up the remains in the cup with the spoon*))
04R-2⌈ (3.0) yea:
⌊ ((*R seems to struggle with her swallowing*))
05N-2((*N gives R another spoon of yogurt*))
06R-2((*R swallows then shakes her head*))
07N-2((*N shakes her head*))

**Extract 2: Task-related interaction: presenting a choice regarding the performance of the task (dyad 4).**
01N-4⌈shall you wash your face and such when you have your sweater on?
 ⌊((*N puts used paper towels in paper bin then turns towards R*))
02R-4yes
03N-4yes ((*N starts removing R's glasses*))

**Extract 3: Task-related interaction: asking about personal experiences or preferences in the task (dyad 3).**
01N-3those are also
02⌈facial creams those two
 ⌊((*points to pots on basin*))
03R-3⌈oh yes
 ⌊((*nods*))
04N-3yes
05(1.0)
06⌈(0.8)
⌊((*puts cream on R's face*))
07N-3but it is this one you like the best?
08R-3⌈°hh°
⌊((*subtle nod*))
09N-3hha

**Extract 4: Task-related interaction: asking about personal issues related to actions in the task (dyad 3).**
01N-3it is dark brown
02R-3((*R nods*))
(1.0)
03N-3have you always been dark?
04R-3(0.5) ⌈no
⌊((*R shakes head*))
05N-3no (0.3) you have had lighter
06R-3⌈h yea
⌊((*nods*))
07N-3Yes

**Extract 5: Non task related interaction (dyad 1): asking about resident's whereabouts last night.**
01N-1⌈have you slept well (0.3) eh lennie?
⌊((*putting away medicine list in cupboard*))
02R-1Na
03N-1⌈what
⌊((*looking at resident then back into cupboard again*))
04R-1(3 syllables (0.2)4 syllables neologistic speech)=
05N-1= ⌈or did you stay up late watching TV?
⌊((*gaze shifting between cupboard and resident*))
06R-1⌈a (4 syllables neologistic speech)
⌊((*nods*))
07N-1was it any good sports?* ((gaze shifting between basin and resident))*
08R-1(10 syllables neologistic) (0.5) ⌈ (2 syllables)
⌊((*nods*))
09(1.0)
10N-1⌈is it a lot ice hockey started yet? (0.5) on the telly?
⌊((*looking at resident*))
11R-1(8 syllables neologistic speech)

Most of the time in the video-recorded interactions was spent on performing activities involved in the main goal of getting the resident ready for the day or night. In those* task-central interactions* the nurses were, for example, focusing on upper body washing, brushing teeth, or shaving. This* task-central interaction* may be performed in silence and without eye contact. However, it may also be accompanied by vocal or nonverbal interaction where the topic of conversation or purpose of the interaction would be directly related to the nursing activity. For example, the nurses would be giving instructions or an account of what they are about to do and why. This may also include a report of how things are going in the activity; see Extract 1 where the nurse (N) in dyad 2 is administering medication in yoghurt with a spoon to the resident (R).

In line 1 the nurse comments on how the task is proceeding. The resident seems to have trouble swallowing and her next turn in line 04 is delayed 3 seconds. When swallowing the last spoonful of yoghurt in line 06, she shakes her head and the nurse acknowledges this by shaking her head too. This type of interaction may also include social interaction in the form of smiles, gaze and touch but no vocal expressions beyond those used to get the nursing task done or commenting on the performance in the task. This main task-central activity continues, with a few exceptions, during the whole time in the video-recordings.

When the nurses were focusing more on the goal of social interaction with the residents, the interaction was usually still* task-related*, but the particular sequence of interaction was not necessary for getting the task done and would often include involvement of the resident as a person with an agency. The nurses would, for example, involve the resident in the performance of the task by presenting a choice as in Extract 2 where the resident in dyad 4 is sitting in the bathroom in front of the mirror and they are about to start washing her upper body.

In line 1 the nurse suggests that the resident keeps her sweater on while washing her face. Although reports on what was going to happen in* task-central interaction* may also be worded as a choice question or a suggestion, the nurses in these* task-related interactions* actually await a response from the resident. In this case the nurse acknowledges the residents response in line 3, before she proceeds with the task by removing the resident's glasses.

These* task-related interactions* may also involve an assessment of the residents' personal preferences or experiences related to the task at hand, as in Extract 3 where dyad 3 is in the bathroom doing upper body care. The nurse has presented the resident with a choice between different types of face cream and the nurse is applying face cream to the resident's face, holding the chosen face cream pot in her hand.

This extract shows that the nurse in dyad 3 has involved the resident in the performance of the task by inviting her to choose type a cream. However, she elaborates on the topic of choice of face cream. First she establishes that the other pots also contain face cream (line 01) and then she asks for a confirmation from the resident that the cream she chooses is the one she prefers (line 04). In this way she makes the interaction more person-centred as she takes the opportunity to not only enable the resident to participate in the task, but to also get to know her a little bit better by asking her about the reason for her choice.

Extract 4 displays an example of care based interaction where the topic relates less directly to the task at hand. Instead by asking about the resident's hair colour the nurse is bringing forward personal issues beyond the task and the present life in the nursing home. In Extract 4 the resident in dyad 3 is sitting in front of the mirror in the bathroom and the nurse is standing behind her brushing her hair.

In line 01 the nurse in dyad 3 is making an assessment regarding the colour of the resident's hair. The resident acknowledges her assessment in line 02 and the nurse proceeds by asking if she always had this hair colour (line 03) and the resident produces a rejection. While the comment is made in the context of the nursing care activity, the nurse is brushing the resident's hair, the topic concerns the quality of the resident's hair in general and at other times and contexts beyond the nursing activity. The nurse in dyad 3 elaborates more on the topic by suggesting that the resident had had lighter hair (05). Her utterance in line 05 is not worded as a question but put as an assertion which the resident affirms in line 06. That is, the nurse shows that she has knowledge about the resident, or at least about issues related to the colouring of hair, and in this they have a shared knowledge and interest. By asking about the resident's previous hair colour when brushing her hair, the nurse is bringing forward personal issues beyond the task and also beyond the resident's present life in the nursing home which potentially makes the care she provides more humanised and person-centred (Dahlberg et al., 2009).

Sometimes, the nurses tended to take the social interaction a step further from the nursing task by addressing more general personal issues with no relation to the ongoing activity. The nurses would, for example, comment on or ask questions about matters in the immediate context or about previous experiences or future plans on behalf of the resident, or talk about their own personal issues, as in Extract 5 where the nurse in dyad 1 is withholding the physical nursing activity and asking the resident about his whereabouts the night before.

In line 01 the N-1 is asking the resident about how he had slept, which is an issue that does not seem to be related to the task at hand where the nurse has just given R-1 his medication. She acknowledges the negative response from N-1 in line 02, but instead of initiating repair on the resident's neologistic utterance in line 04 she keeps up the flow of the conversation by her latched-on suggestion that he stayed up late watching TV (line 05). The one second pause after R-1's longer neologistic utterance in line 08 indicates that she is having trouble understanding him, but again, instead of initiating repair, she asks another question about the sports on TV (line 10). This and her question in line 07 about whether there had been any good sports on TV allow her to demonstrate for the resident that she has knowledge about his personal preferences as he often stays up late watching sports on TV.

Although the nurse at first seems to still be somewhat engaged the nursing task, as her gaze is shifting between first the cupboard and the resident (lines 03 and 05) and then the basin and the resident (line 07) she has withheld the actual physical nursing activity. In line 10, her gaze is focused on the resident and their social interaction when she elaborates on the topic by asking about the ice hockey season.

### 3.4. Results from Analysis of Distribution in Time

**Figure 1:**
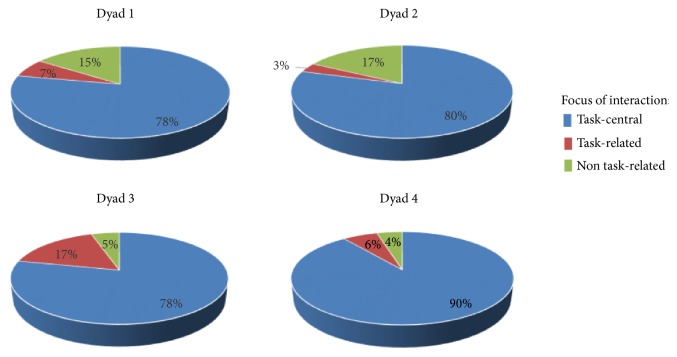
The distribution in time of the three different interactional foci in the four dyads.

The results from the analysis of distribution in time of the three types of interaction showed that the main proportion of the interaction in the activity was* task-central* and focused on getting the routine task done. In all four dyads this represented between 78 and 90% of the time. However, despite the fact that all four of the residents had similar problems due to their severe aphasia, there was some variation between the dyads in terms of how much of the time was focused on task-central, task-related, or non task-related interaction; see Figure 1.

The distribution of time spent with the different types of interactional foci may be related to patterns in the dyads' communicative interaction. Most time was spent in* task-central interaction* by dyad 4, where it amounted 90%. The resident in this dyad was in general quite passive in the interaction and the nurse-initiated topics were usually related to the routine care task. In dyad 4 only 4% of the time in interaction had a focus on* non task-related *issues.

In dyad 2, where the resident was the one who most often initiated interaction and produced longer utterances as much as 17% of the time was spent on* non task-related interaction, *which is similar to the distribution in dyad 1 (15%). A large part of this interaction may be related to the fact that the nurses in both dyads 1 and 2 tended to keep up the flow in the conversation by telling the residents about their own activities and experiences since they last met. In dyad 3, 17% of the interaction was instead* task-related*, which may be explained by the fact that the resident in dyad 3 had a personal interest in the use of different beauty products and this was utilized by the nurse to facilitate the social interaction.

## 4. Discussion 

The qualitative analysis in this study showed that the interactional foci in the studied routine care tasks were affected by both a goal aimed at getting the resident ready for the day/night and a goal aimed at social interaction. The nurses' aim to interact socially with the residents could be described on a continuum of different types of interaction involving different topics, which could be more or less task-related. As all four residents had severe aphasia, interaction more or less related to the task at hand is easier to accomplish than non task-related communication as the “here and now” focus and the artefacts used supports the participants' mutual understanding. Still, the quantitative analysis showed that although the activities focusing on getting the physical routine care task done dominated the time spent in the main activity, there was also quite some time spent in interacting socially with the residents.

The distribution in interactional foci is in line with the results in Williams and colleges study [17]. If merging the categories used by Williams and colleagues that was related to the nursing task, 71% of the utterances concerned task-central issues and 29% were more personal, psychosocial in nature. However, Williams and colleagues coded each utterance of the nursing staff, while in the current study the time allowed for the residents' contributions on the topic is also included in the analysis, which makes comparisons difficult.

The different types of goals in the main activity entail that the participants need to take on different roles. A person's social identity may be considered a flexible resource that can change to adapt to demands of the situation [51]. Facilitating conversations on a topic that shift the focus of the interaction away from the nurse's care, allows the participants to take on another role and present another social identity. Doing this is also a way of facilitating person-centred care where the resident as an individual with personal experiences and traits is recognised and acknowledged [7, 8]. However, the coding of interaction as task-related or non task-related does not say anything about whether the interaction concerns what is considered as important or personal for a specific resident.

From the quantitative analysis it could be concluded that the adaptation of the nurses to the residents' individual background factors seemed to have a greater impact on the distribution of foci of interaction than the type of activity or the residents' ability to communicate. The nurses in dyads 1 and 2 both managed the more personal, less task-central social interaction by dominating the floor, avoiding long repair sequences and sharing information about their own lives. The inherent time constraints in the activity probably impose a certain amount of stress in both the nurses and the residents [14]. This may lead to an avoidance of extended repair sequences when mutual understanding is compromised, despite knowledge about how to repair. Still, these nurses did not avoid social interaction. The nurse in dyad 1 used her knowledge of the resident's former life as well as present interests and family to support communication with the resident. In doing this, the social interaction could be more fluent and maintained over time. The nurse in dyad 3 also used her knowledge of the resident's personal interests in the activity for task-related interaction which in that way became more personal. In dyads 1-3, the nurses demonstrated an ability to use their experiences of how to adapt the communication to the needs of the residents, allowing the residents to do their best as conversation partners using the means they had to communicate despite their severe aphasia.

Regarding dyad 4, we cannot tell from the data whether the focus on task-central and task-related issues in the interaction was due to a reluctance by the nurse or the resident to take on the role of conversation partner in more personal social interaction. The nurse had experience of working with people with communication disorders and evidence suggests that both nursing staff and residents in long-term care in general do want to communicate on more personal topics, beyond the caring tasks [14–17]. However, it may be that personal traits of either the nurse or the resident were factors preventing more verbal social interaction. Or perhaps neither of them was interested in communicating on more personal topics during the video-recordings. Still, the resident would of course be dependent on the nurse's facilitating behaviour to be able to do this if she wanted to. We might hypothesise that nursing staffs' attitudes and knowledge about aphasia are an important factor, particularly given that it is usually nurses who initiate vocal communicative interaction and decide on what topics to elaborate when the residents' initiate communication [14, 15]. There is evidence that communication partner training can facilitate communication for people with aphasia and their communication partners [52] and many national stroke guidelines now recommend communication partner training for health care providers [53, 54].

The coding scheme used in the present study was based on qualitative analysis of the data. Still, it has been questioned whether it is possible to make a distinction between a task-central interaction or more relational, affective, social talk [55]. Nursing staff do report on how they sometimes use relational or affective communication in the purpose to accomplish a task [23]. Qualitative analysis of interaction with residents with dementia in long-term care has also shown that the involvement of and adaptation to the residents' personal concerns may facilitate the completion of a physical care tasks [37]. The complexity of interaction between residents or patients and health care providers or significant others [56] calls for analysis that considers both external and internal sequential contextual as well as personal factors.

## 5. Conclusions

The interactional foci in routine care tasks in long-term residential care may be more or less task-related, but this study also provides evidence that nurses can spend quite a lot of time interacting socially in a personal manner with residents with severe communication disorders in everyday routine tasks. The methods used in this paper, combining a discourse analysis influenced by CA and ACA allowed the analysis to include both internal and external contextual factors, which is important in the exploration of person-centred care. The results show that variations in amount of time spent in social interaction may be more related to personal factors than to severity of communication disorder or type of activity. This has important implications for clinical practice as well as research. Although it still remains to find out how to best study, define, and conceptualise person-centred care, contextual and personal factors need to be considered. Furthermore, residents with aphasia and nursing staff in long-term care may benefit from communication partner training and a personalized approach in general to safeguard quality care.

## Figures and Tables

**Table 1:** Description of nurses (N) and the residents with aphasia (R) including measures of comprehension in Token test [48] and verbal fluency [49] and amount of data from each dyad.

**Nurses**	**N-1**	**N-2**	**N-3**	**N-4**
Sex/Age	F/55	F/55	F/40	F/36
Time working with R	3 years	19 months	9 months	1 year
Education, in years	11	12	12	12

***Residents with aphasia***	***R-1***	***R-2***	***R-3***	***R-4***

Sex/Age	M/72	F/91	F/82	F/93
Type of aphasia^a^	Severe, global aphasia	Severe, global aphasia	Severe, global aphasia	Severe, global aphasia
Time post onset	8 years	17 months	10 months	12 months
Time in residential care facility	3 years	19 months	12 months	12 months
Score in Token Test	69	97	36	68
(max: 261)				
Score in word fluency tasks	0/0/0	0/0/0	0/0/0	0/0/0
Amount of analysed data (in minutes)	70	50	90	50

^a^Aphasia type according to the Boston classification system.

**Table 2:** Key to transcription symbols.

((nodding))	Non-verbal activity within double brackets
⌈ yes	Simultaneous verbal or non-verbal activities
⌊ ((nods))	
(0.7)	Numbers in parentheses indicate silence in tenth of second
no:	A colon indicates an extension of the sound or syllable it follows
°no°	Degree signs indicate a passage of quiet talk

## Data Availability

The video data used to support the findings of this study are restricted by the Regional Ethical Review Board of Västra Götaland in Sweden in order to protect participant privacy. Data are available from the corresponding author for researchers who meet the criteria for access.
